# Nomenclature for endogenous retrovirus (ERV) loci

**DOI:** 10.1186/s12977-018-0442-1

**Published:** 2018-08-28

**Authors:** Robert J. Gifford, Jonas Blomberg, John M. Coffin, Hung Fan, Thierry Heidmann, Jens Mayer, Jonathan Stoye, Michael Tristem, Welkin E. Johnson

**Affiliations:** 1 MRC-University of Glasgow Centre for Virus Research, Glasgow, UK; 2 Department of Medical Sciences, Uppsala University, Uppsala, Sweden; 3 Department of Molecular Biology and Microbiology, Tufts University, Boston, MA USA; 4 Department of Molecular Biology and Biochemistry and Cancer Research Institute, University of California, Irvine, CA 92697 USA; 5 Department of Molecular Physiology and Pathology of Infectious and Endogenous Retroviruses, CNRS UMR 9196, Institut Gustave Roussy, 94805 Villejuif, France; 6 Department of Human Genetics, Center of Human and Molecular Biology, Medical Faculty, University of Saarland, Homburg, Germany; 7 The Francis Crick Institute, Mill Hill Laboratory, The Ridgeway, Mill Hill, London, UK; 8 Imperial College London, Silwood Park Campus, Buckhurst Road, Ascot, Berkshire, SL5 7PY UK; 9 Biology Department, Boston College, Chestnut Hill, Massachusetts, 02467 USA

**Keywords:** Retrovirus, Nomenclature, Endogenous, Taxonomy, Classification

## Abstract

Retroviral integration into germline DNA can result in the formation of a vertically inherited proviral sequence called an endogenous retrovirus (ERV). Over the course of their evolution, vertebrate genomes have accumulated many thousands of ERV loci. These sequences provide useful retrospective information about ancient retroviruses, and have also played an important role in shaping the evolution of vertebrate genomes. There is an immediate need for a unified system of nomenclature for ERV loci, not only to assist genome annotation, but also to facilitate research on ERVs and their impact on genome biology and evolution. In this review, we examine how ERV nomenclatures have developed, and consider the possibilities for the implementation of a systematic approach for naming ERV loci. We propose that such a nomenclature should not only provide unique identifiers for individual loci, but also denote orthologous relationships between ERVs in different species. In addition, we propose that—where possible—mnemonic links to previous, well-established names for ERV loci and groups should be retained. We show how this approach can be applied and integrated into existing taxonomic and nomenclature schemes for retroviruses, ERVs and transposable elements.

## Background

**Fig. 1:**
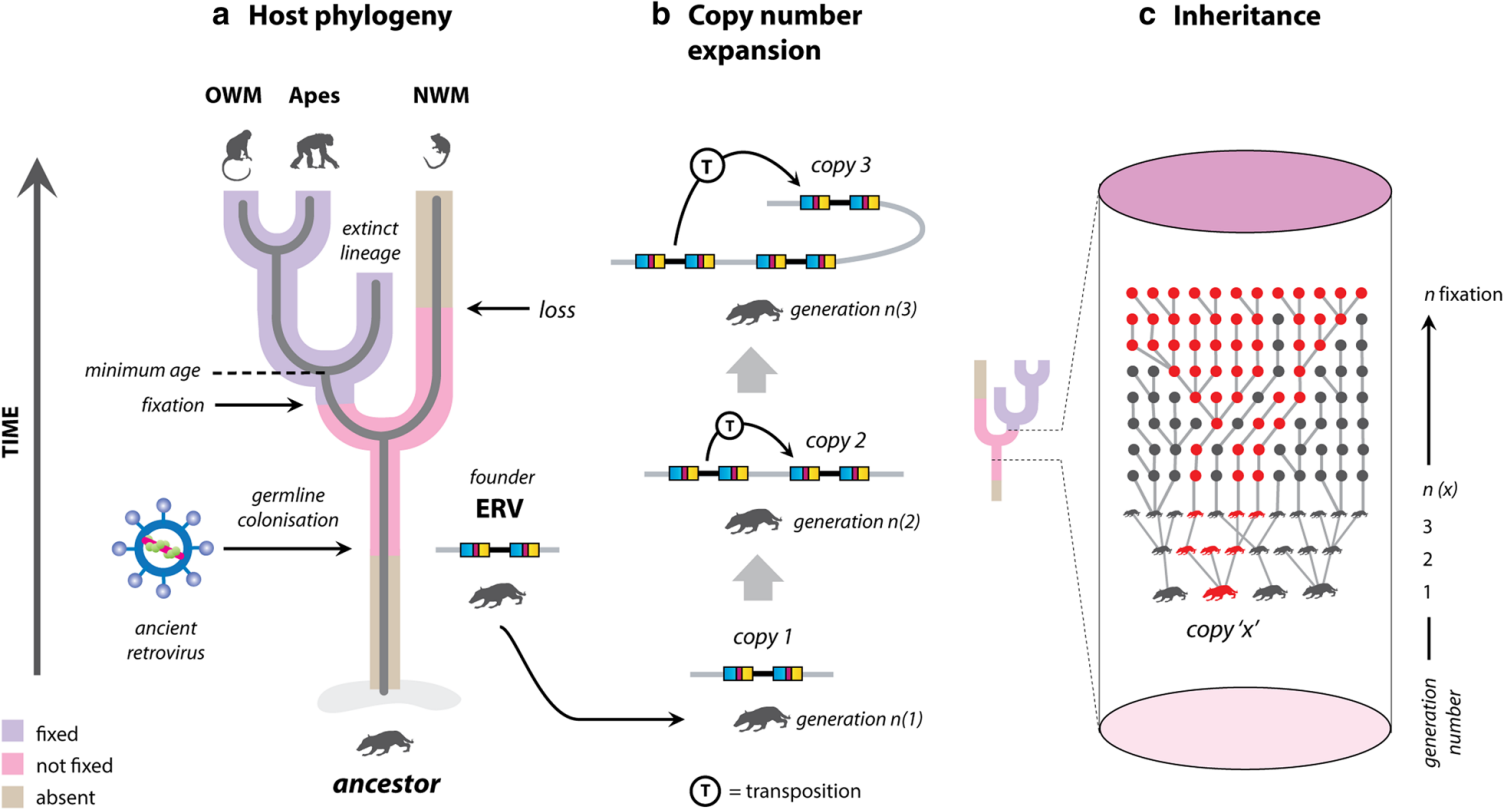
Retroviral genome invasion and the fate of endogenous retrovirus (ERV) loci in the germline. The three panels show schematic diagrams illustrating how the distribution of ERVs is influenced by **a** host phylogeny; **b** activity of ERV lineages within the gene pool; **c** patterns of ERV locus inheritance within populations of host species. Panel **a** shows how ERV lineages originate when infection of an ancestral species by an ancient retrovirus causes a ‘germline colonisation’ event in which a retroviral provirus is integrated into the nuclear genome of a germline cell that then goes on to develop into a viable organism. This ‘founder’ ERV provirus can subsequently generate further copies within the germline (panel **b**). The fate of individual ERV loci is determined by selective forces at the level of the host population. Most ERV loci are quickly eliminated from the germline via selection or drift. However, some may increase in frequency from one host generation to the next, to the point where they become genetically ‘fixed’—i.e. they occur in all members of the species. The schematic in panel **c** illustrates this in a simplified way, showing an ERV locus (copy x) becoming fixed in over several host generations. As shown in panel **a**, fixed ERV loci persist in the host germline as ‘footprints’ of ERV activity, and the identification of orthologous ERV loci in multiple species indicates that those species diverged after the ERV was inserted. Thus, when host divergence dates have been estimated, they can be used to infer minimum ages for orthologous ERV loci. Importantly, extinction of host lineages eliminates swathes of ERV loci. In some rare cases, however, their sequences may still be recoverable (e.g. see [79]). Abbreviations: *ERV* endogenous retrovirus, *NWM* New World monkeys, *OWM* Old World monkeys

Retroviruses (family *Retroviridae*) are characterized by a replication cycle in which the viral RNA genome is reverse-transcribed and integrated into the nuclear genome of the host cell. The principal determinants of the retroviral replication cycle are the enzymes reverse transcriptase (RT) and integrase (IN) [1]. These enzymes allow the conversion of single stranded viral RNA into double-stranded DNA, followed by integration of viral DNA into the nuclear genome of the infected cell to form the ‘provirus’. As a chromosomal insertion, the integrated provirus has a life-long association with the infected cell, and survives as long as that cell (or its progeny). When integration occurs in a germ cell (i.e. gametes or early embryo), the resultant provirus can be vertically inherited as a host allele (see Fig. 1). Such a provirus is called an endogenous retrovirus (ERV). Unless silenced or inactivated (e.g., by methylation [2] or mutation), ERV proviruses retain the potential to give rise to additional germline copies—either by infection of, or retrotransposition within further germ cells [3–5]. Selective forces operating at the level of the host population determine the fate of individual ERV loci. By far the most likely outcome for any newly generated ERV locus is that it will be purged from the gene pool. Despite this, however, vertebrate genomes typically contain thousands of ERV loci that have been genetically ‘fixed’—i.e. they occur in all members of the species [6].

Studies over recent years have revealed the profound impact that ERVs have exerted on vertebrate evolution. For example, more of the human genome (~ 8%) is made up of the remnants of past retroviral infections than of sequences encoding the proteins necessary for life (~ 1–2%) [7]. Moreover, ERVs are not—as was once believed—mere ‘junk DNA’—some encode intact proteins that have been co-opted or exapted to perform physiological functions in host species, and even ERVs that are relatively degraded in terms of their coding capacity can perform important functions as components of gene regulatory networks [8–13].

ERV sequences also provide a unique source of retrospective information about retroviruses that circulated millions of years ago, and can therefore be used to explore the long-term history of evolutionary interaction between retroviruses and their hosts [14, 15]. Until quite recently, most investigations of this nature have of necessity been theoretical or comparative, but in recent years ‘investigators have utilized gene synthesis to ‘repair’ the mutated genes of ERVs and study their biological properties in vitro [16–25].

New vertebrate genome sequences are becoming available for study on an almost daily basis, providing a deluge of novel ERV data to drive further investigations of ERVs. There is therefore an urgent need for a unified system of nomenclature for ERV loci, not only to assist genome annotation, but also to facilitate research on ERVs and their impact on the genome biology and evolution of host species.

## Insights into ERV biology in the genomic era

Modern genomics has allowed investigations of ERVs across a wide range of vertebrate whole genome sequences [26]. Together, these have provided a number of important insights into the general biology of ERV lineages that should be taken into consideration when constructing a nomenclature system.

Firstly, phylogenetic studies in humans and other species have shown that the multitudes of ERV sequences found in vertebrate genomes derive from a relatively small number of initial founder events [27, 28], and that distinct vertebrate lineages contain characteristic sets of ERVs that reflect their specific histories of; (1) retroviral germline invasion; (2) ERV copy number expansion; (3) and ERV locus fixation (see Fig. 1). However, establishing precisely the number of distinct retroviral germline invasion events that have occurred in the evolution of a host lineage is difficult. Significant germline invasions by retroviruses can presumably occur without any ERVs being fixed in descendant species, and even those ERV groups that do get fixed may be comprised entirely of partial and/or low copy number sequences that are problematic to detect. Moreover, even for the subset of ERVs that are detectable, phylogenetic approaches may not allow the number of separate invasion events to be determined with confidence—particularly when multiple invasions involving relatively similar viruses have occurred in the distant past. For example, estimates for the number of distinct germline invasion events that gave rise to the ERVs found in the human genome vary widely, from ~ 34 to ~ 80 [10, 73].

**Fig. 2:**
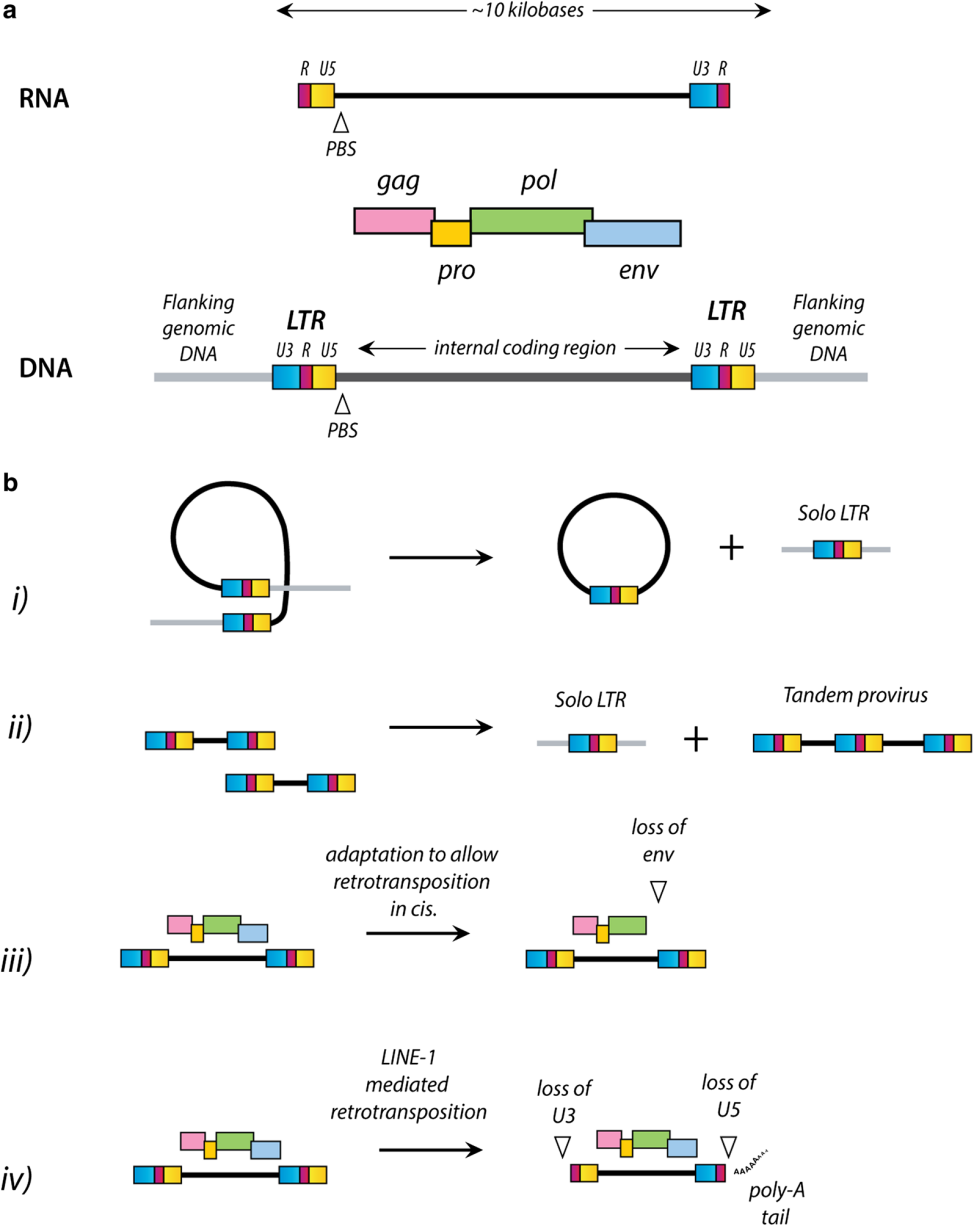
Genomic structure of ERV sequences. Panel **a** shows a schematic representation of a generalised retroviral provirus. The four coding domains found in all exogenous retroviruses are indicated. The precise organization of these domains varies among retrovirus lineages, and some viruses also encode additional genes. The long terminal repeat (LTR) sequences are comprised of three distinct subregions that are named according to their organization in the genomic RNA: unique 3′ region (U3), repeat region (R), and unique 5′ region (U5). Panel **b** shows a schematic representation of processes that modify ERV sequences. (1) Recombination between the two LTRs of a single provirus resulting in the formation of a solo LTR. (2) Recombination between the 3′ and 5′ LTRs of a given provirus leading to a tandem duplicated provirus. (3) Adaptation to intracellular retrotransposition, resulting in the loss of the envelope gene. (4) LINE1-mediated retrotransposition, resulting in loss of the 5′ U3 sequence, and the 3′ U5 sequence. Variants with larger 5′ truncations may also occur. Poly-A tails at the 3′ end and L1-typical target site duplications flanking the retrotransposed sequence are usually found for these forms. Figure partly adapted from [80]

Secondly, it is clear from genomic studies that the vast majority of ERVs no longer encode functional proteins. Retroviral proviruses typically possess three principal coding domains (*gag*, *pol* and *env*), flanked at either side by long terminal-repeat sequences (the 5′ and 3′ LTRs) that are identical at the time of integration [29] (Fig. 2). A non-coding sequence containing a tRNA-specific primer-binding site (PBS) is usually present between the end of the 5′ LTR and the first codon of the *gag* gene. Without the purifying selection provided by replication, however, ERV sequences undergo mutational decay. Frequently, internal coding sequences are completely deleted through recombination between 5′ and 3′ LTRs, leaving behind a ‘solo LTR’ [30]. Indeed, solo LTR numbers are typically orders of magnitude more common than loci containing internal coding regions [31]. Other rearrangements of ERV genomes can also arise through processes such as LINE1-mediated retrotransposition, recombination, and deletion (Fig. 2b) [3]. Recombination can generate a diversity of ‘mosaic’ ERV forms [6], and can lead to genes and LTR sequences being ‘swapped’ between retroelement lineages [32].

Finally, comparative genomic studies have shown that in many cases, homologous ERV sequences are present at the same genomic locus in multiple species genomes. Since retroviral integration—while not random—is not site-specific [33, 34], such ‘orthologous’ ERV loci can be assumed to have been generated before the species they are found in diverged. Thus, if host divergence dates are known, they can be used to infer minimum ages to be inferred for individual ERV loci, and by extension the founding colonization events that generated ERV lineages [35]. In higher primates, for example, comparative studies show that most integration events are extremely ancient, having occurred after the separation between New World monkeys (Platyrrhini) and Old World monkeys (Catarrhini) but before the split between Old World monkeys and hominoids (*Hominoidae*) around 30–45 million years ago (Mya) [36]. It should be noted, however, that fixed ERV loci may significantly predate the divergence times of the host species they occur in. Furthermore, as shown in Fig. 1, fixed ERV loci can be much younger than the ERV lineage they belong to, and due to different patterns of inheritance in descendant hosts, ERVs can end up being fixed in one set of descendant species, and lost from another.

## Existing ERV nomenclature schemes and history of their development

Existing nomenclature systems for ERVs have developed in a haphazard manner reflecting their history of discovery. ERVs were first discovered in the 1960s by virtue of the genetically controlled expression of viral antigens of replication-competent ERVs in chickens and mice [37]. These viruses were closely related to exogenous oncogenic viruses, prompting a decades long search for disease-associated ERVs in other species, especially man [38, 39]. Infectious human counterparts, however, have remained elusive.

Laboratory techniques employed to identify ERVs have included virus isolation by co-cultivation with cells from a variety of species [40], hybridization under low stringency conditions with retroviral probes followed by cloning [41, 42], and PCR with primers directed to conserved regions of RT [42–46]. These studies formed the initial context of ERV nomenclature schemes, but in more recent years, ERV nomenclature has been increasingly influenced by in silico mining of vertebrate genome sequences, based either on sequence similarity or predicted features of proviruses such as nearby LTRs.

Originally, endogenous proviruses were named after the most closely related exogenous retrovirus, such as murine leukemia virus (MLV), as well as subgroups, like xenotropic MLV (XMV) [47]. A common approach to naming ERVs in different species has been to add one or two letters before the designation ERV to indicate the species in which they were initially identified; thus, HERV indicates an ERV first seen in human DNA, and MERV or MuERV implies one originally found in the genomes of murine species [e.g. house mouse (*Mus musculus*)]. HERVs have been further classified on the basis of the tRNA that binds to the viral primer binding site (PBS) to prime reverse transcription (see Fig. 2a). Hence HERV-K implies a provirus or ERV lineage that use a lysine tRNA, no matter their relationship to one another. In some cases the PBS sequence was not available when novel elements were first discovered leading to the names based on neighboring genes (e.g. HERV-ADP [48]), clone number (e.g. HERV-S71 [49]), or amino acid motifs (e.g. HERV-FRD [42]). Additional designations based on the probe used for cloning, and sub-divisions based on sequence identity or phylogenetic reconstructions, have also been used [50].

The somewhat arbitrary manner in which these nomenclatures have evolved has created a number of anomalies. The first concerns the use of the initial letter(s) to designate species of origin. This presents difficulties with proviruses that were integrated prior to the divergence of their host species. Many of the ERVs present in humans and chimpanzees fall into this category—thus related proviruses in both species genomes can end up with quite different names (e.g. HERVxxx and CERVyyy) despite the fact that proviruses in the two species will be more closely related to one another (identical at the time of integration) than their paralogous siblings within the same phylogenetic grouping. This problem becomes even more acute when considering specific proviruses shared among multiple species (i.e., when the same integrated provirus has been inherited by two or more descendant species). A further difficulty arises when what would appear to be the generic name for ERVs from one species becomes the trivial name for a discrete lineage of proviruses within that species, as has occurred with the MLV-related PERVs (porcine endogenous retroviruses) of pigs [51].

The use of tRNA primer specificity as a basis for sub-classification is problematic because there are a number of instances where this sequence does not reflect the overall relationship between distinct ERV lineages. For example, the HERV-K(HML-5) group appears to use a tRNA_Met_ as primer while the other HERV-K lineages use tRNA_Lys_ [52]. Even very recently integrated proviruses, such as endogenous MLVs, can be found to use different tRNA primers. The frequent convergent evolution implied by these examples, and the limited number of tRNAs available, makes primer usage an unsuitable basis for retroviral taxonomy.

At the level of individual ERV lineages, it is necessary to distinguish among specific proviruses at discrete chromosomal locations (i.e. between different but related ERV loci), and several different systems have developed for this purpose. Most commonly, individual proviruses are simply numbered; e.g. as *Xmv1*, HERV-K 108, etc. In the case of HERVs, some investigators have chosen to use cytogenetic designations to distinguish among related proviruses [53, 54], as in HERV-K 11q22 (located on the q-arm, chromosomal band 22, of human chromosome 11). The need for this kind of locus-level ERV annotation is far more urgent now that large numbers vertebrate genomes have been sequenced. Indeed, in genomes that have been sequenced to a high degree of coverage, it is now feasible to identify and annotate the majority of ERVs using purely in silico approaches.

The most comprehensive source of repetitive element annotations is REPBASE [55]. REPBASE annotations, which include but are not limited to ERVs, are based on sequence similarity to a set of consensus elements. As such, the naming conventions used within REPBASE may not necessarily reflect phylogenetic relationships between ERVs. Also, REPBASE annotations distinguish LTRs and internal regions, but do not provide any further breakdown of the genomic features found within ERV proviruses. Software tools have also been developed specifically to assist in the identification and characterization of ERVs (for instance, see [56–58]), and these, more focused systems can be used to map ERVs to a fine scale of detail, demarcating genes, protein domains, and functional RNA sequences [6, 59]. Unfortunately, however, there is currently no straightforward way to link the ERV annotations generated by distinct systems with one another, or with the taxonomic groupings of ERVs that have been defined in broad-based phylogenetic studies [27, 28, 45, 60–62].

## Integrating ERV classification with retrovirus taxonomy

A further problem is aligning ERV classification—which so far has been derived in large part from systems of repetitive element annotation—with retroviral taxonomy as agreed by the International Committee for Virus Taxonomy (ICTV). The *Retroviridae* family is grouped into the order *Ortervirales* (retro-transcribing viruses) [63], and comprises two sub-families, *Orthoretrovirinae* (orthoretroviruses) and *Spumaretrovirinae* (spumaviruses or ‘foamy viruses’). *Spumaretrovirinae* is currently a monogeric subfamily, whereas the *Orthoretrovirinae* comprises six exogenous genera. Endogenous representatives have now been identified for the majority of retroviral genera (Table 1). Some of these ERVs group robustly within the diversity of exogenous representatives in phylogenetic trees. Others group basal to contemporary isolates, but exhibit genomic or phylogenetic characteristics that argue for their inclusion within a particular genus (e.g. the presence of characteristic genomic features such as accessory genes and nucleotide composition biases) [64–66].

**Table 1:** Retroviral genera and their endogenous representatives

Genus	Type species	Endogenous representative^a^	
*Alpharetrovirus*	ALV	ALV	[37]
*Betaretrovirus*	MMTV	MMTV	[74]
*Gammaretrovirus*	MLV	MLV	[75]
*Deltaretrovirus*	HTLV-1	MinERVa	[66]
*Epsilonretrovirus*	WDSV	*none* ^b^	
*Lentivirus*	SRLV-A	RELiK	[64]
*Spumaretrovirus*	SFV	SloEFV	[65]

*ALV* avian leukosis virus, *MMTV* mouse mammary tumour virus, *MLV* murine leukemia virus, *HTLV* human T cell leukemia virus, *WDSV* walleye dermal sarcoma virus, *SRLV-A* small ruminant lentivirus A, *SFV* simian foamy virus, *MinERVa Miniopterus* endogenous deltaretrovirus, *RELiK* rabbit endogenous lentivirus K, *SloEFV* sloth endogenous foamy virus

^a^First reported endogenous representative shown, with citation

^b^No ERVs have been identified that group robustly within the *Epsilonretrovirus* genus. However, distantly related, ‘epsilon-like’ elements have been described, such as the MER65/HERV-Lb elements found in the human genome [6, 76–78]

However, most ERV lineages are more problematic to place in current taxonomic systems, and as a consequence, many have become known by the relatively arbitrary names they have been assigned within repetitive element classification systems. In these systems, ERVs form part of a larger assemblage of LTR-retroelements [55, 67, 68] characterised by their “paired LTR” structure. TE classification systems conventionally group ERVs into three ‘classes’ (I, II and III), based on relatedness to the exogenous *Gammaretrovirus*, *Betaretrovirus* and *Spumaretrovirus* genera respectively. Individual ERV lineages (i.e. groups of ERVs that are assumed to derive from a single germline invasion event) have historically been referred to as ‘families’. This is problematic as the terms ‘class’ and ‘family’ have specific, taxonomic meanings and their use in this context is incompatible with existing retroviral taxonomy.

Taxonomy should ideally follow phylogeny [69]. Since the overwhelming evidence from genomic studies indicates that endogenous retroviruses derive from ancient exogenous retroviruses, integration of ERVs into retroviral classification schemes is both feasible and logical, following this principle. Any novel system of classification for ERVs should therefore take into account the phylogenetic relationships of ERVs to exogenous viruses. In addition, it seems likely that integration of ERV nomenclature with exogenous retroviral taxonomy will require the definition of new groups to represent lineages that existed as exogenous retroviruses in the past but now exist only as ERV “fossils” (i.e., extinct lineages).

## ERV nomenclature proposal

It is clear that a standard system of nomenclature is required. Such a system would greatly facilitate communication and reproduction of results. For example, it could be used to provide unambiguous lists of loci in methods sections of manuscripts, or for the purposes of reproducing or comparing results of different studies. Ideally, a nomenclature system would provide a stable foundation for the development of increasingly accurate and finely detailed annotations. In addition, it could be used to nurture the establishment of a unified taxonomic system for retroviruses and ERVs.

**Fig. 3:**
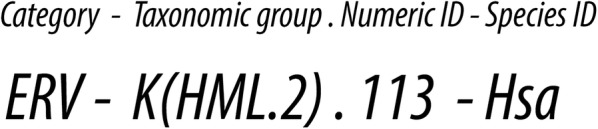
Proposed ERV ID structure. The proposed ID consists of three components separated by hyphens. The second component consists of two subcomponents, separated by a period, that identify (1) the group the ERV belongs to, and (2) the unique numeric ID of the locus. The third component identifies the species or species group in which the element(s) being referred to occur

We therefore propose that ERV loci be assigned standard, unique IDs composed of three elements, each separated by a hyphen, as shown in Fig. 3. The first element is a classifier that identifies the element as an ERV. The second element is itself comprised of two subcomponents—one denoting the lineage of retroviruses that the ERV belongs to, and the second being a numeric ID that uniquely identifies the specific ERV locus within that taxonomic group. The third element identifies the host lineage in which the ERV insertion occurs. The host lineage component may specify a species (i.e. we suggest using well-established abbreviations, such as HomSap for *Homo sapiens*). Alternatively, a higher taxonomic rank may be used to refer to the entire set of orthologous insertions that occurs in an order, family or genus. Examples of how these IDs would be applied to specific ERV loci are shown in Table 2.

**Table 2:** Application of the proposed nomenclature to example ERV loci

Example description	Locus ID
ERV-L insertion identified in all eutherian mammals^a^	ERV-L.1-*Eutheria*
Human copy of ERV-L.1-*Eutheria*	ERV-L.1-*Homo sapiens*
	ERV-L.1-HomSap*
	ERV-L.1-Hsa*
	L.1-Hsa**
HERV.K (HML2) 113	ERV-K(HML2).113-*Hsa*^b^
Chimpanzee ortholog of HERV.K (HML2) 113	ERV-K(HML2).113-Ptr
All copies of HERV.K (HML2) 113 found in great apes (*Hominidae)*	ERV-K(HML2).113-*Hominidae*
Human copy HERV-K(HML2) 4q35.2	ERV-K(HML2).4352-*Hsa*^c^
Polytropic murine leukemia virus ERV 1 (Pmv-1) in mouse	ERV-Pmv.1-Mus musculus
Xenotropic murine leukemia virus ERV 8 (Xmv-8) in mouse	ERV-Xmv.8-Mmu
Mouse mammary tumour virus (MMTV) locus 9 (Mtv9)	ERV-MMTV.8-Mmu
Xmv-8 in inbred mouse strain C57L	ERV-Xmv.8-Mmu.C57L
Copy 2 of rabbit endogenous lentivirus K (RELiK) in rabbit	ERV-RELiK.2-*Oryctolagus cuniculus*
	ERV-RELiK.2-OryCun*
Copy 2 of rabbit endogenous lentivirus K (RELiK) in hare	ERV-RELiK.2-*Lepus europaeus*
	ERV-RELiK.2-LepEur*
	RELiK.2-OryCun**
Macaque copy #183 of an unclassified Betaretrovirus-like virus	ERV-AB.183-*Macaca mulatta*
Peregrine falcon copy #25 of avian ‘Betaretrovirus-like lineage 3′	ERV-AB3.25-*Falco peregrinus*
Use of trailing element to indicate alternative alleles of a polymorphic insertion	ERV-K(HML2).113-Hsa.a^d^
	ERV-K(HML2).113-Hsa.b^d^
Use of trailing element to indicate alternative genome structures of a polymorphic insertion	ERV-K(HML2).113-Hsa.provirus^d^
	ERV-K(HML2).113-Hsa.LTR^d^

*Alternative versions using an abbreviation to designate the host species component of the ID

**A shorter form of the ID can be used when it is clear from the context—or from the lineage component of the ID—that an ERV is being referred to

^a^For reference, see [35]

^b^We propose that where established numeric IDs are already in use, they should be preserved, as is the case for many representatives of the well researched HERV-K(HML2) lineage

^c^In this example, an ID is assigned to an ERV locus that has only previously been referred to via its cytogenetic location—a numeric ID is therefore proposed that preserves a mnemonic link to this cytogenetically-based identifier, without preserving the information about cytogenetic location. This follows a principle of our proposal wherein the numeric ID component of the overall ERV ID can retain mnemonic links to previous IDs, but all auxiliary information associated with ERV loci is obtained from a database via a unique ID, rather than encoded into the ID itself

^d^However, where it aids discussion such information can be appended to the ERV ID stem (e.g. to distinguish distinct alleles and genome structures)

## Applying the proposed ERV nomenclature in practice

There are a number of contingencies pertaining to way that each of the individual elements within the ID is defined. Firstly, only sequences that disclose robust phylogenetic evidence of having been directly derived from an exogenous retrovirus should receive the classifier ‘ERV’ in the first ID element. Thus, loci belonging to the ancient mammalian lineage ERV-L would be included (even though none of the canonical ERV-L sequences encode an *env* gene) because the ERV-L RT has been shown to group robustly within the diversity of the family *Retroviridae* [70]. By contrast, other LTR-retroelements that do not disclose an unambiguous link to retroviruses are excluded. These include, for example, the mammalian apparent retrotransposon (MaLR) elements, which are comprised of LTR-bounded internal sequences containing little or no similarity to retroviruses. Initially, the ‘ERV’ classifier should be reserved for clearly proviral elements that contain recognisable coding domains in their internal regions, and can be placed within a phylogeny of elements that can itself be placed within the *Retroviridae* family. Subsequently, solo LTR loci can be incorporated if: (1) they are allelic variants, and some proviral alleles also occur at the same locus; (2) they fall within a clade of LTR elements that is demonstrably associated with a particular lineage of ERV proviruses.

**Fig. 4:**
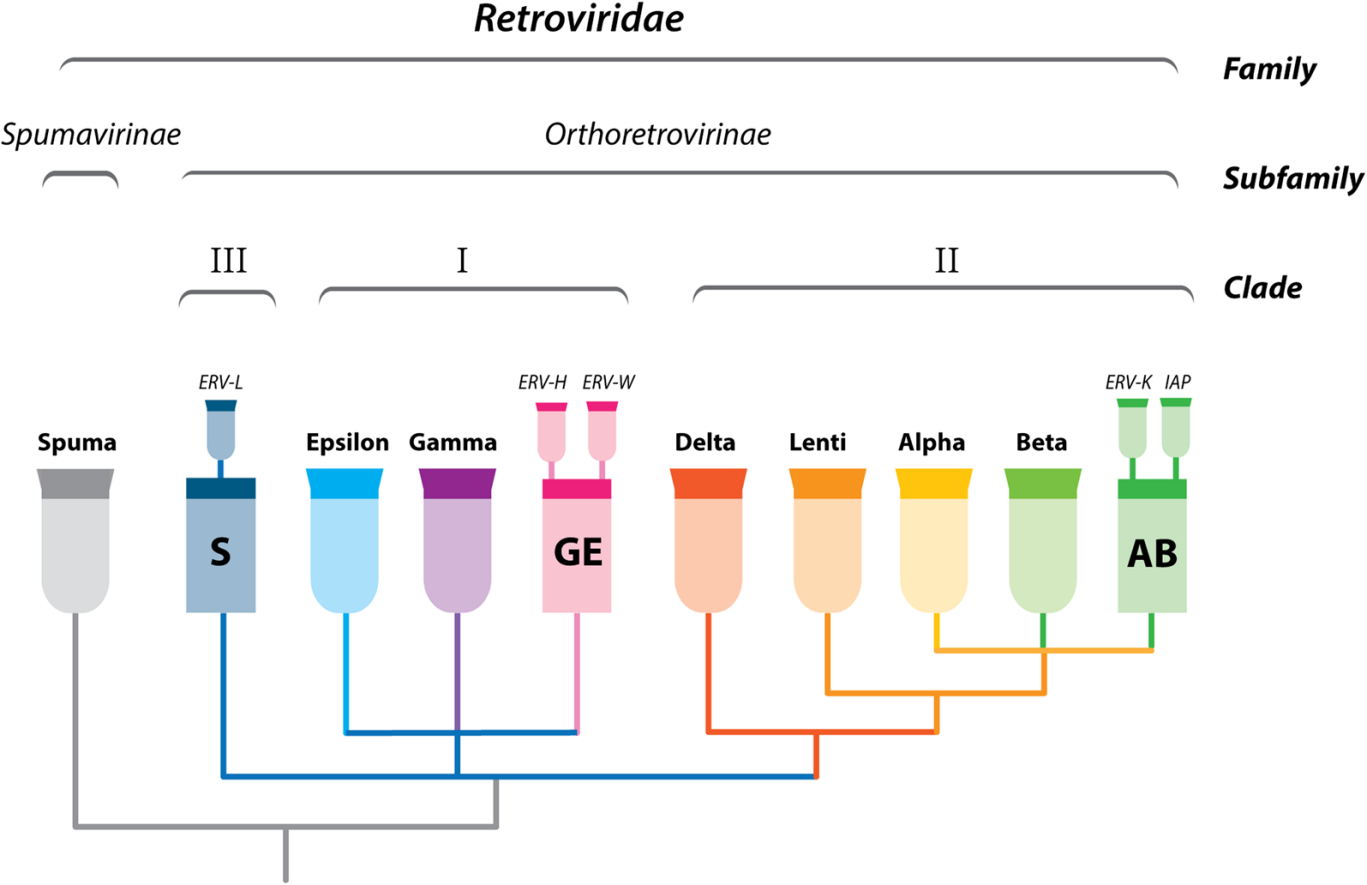
Schematic phylogeny illustrating the basis for a unified ERV and retrovirus taxonomy. The top two brackets indicate taxonomic groupings. The ‘clade’ level reflects three major divergences in orthoretroviral reverse transcriptase genes [71]. The seven officially recognised genera are shown as coloured goblets at phylogeny tips. In addition, three placeholder groups are shown: *Spumavirus*-related (S), *Gammaretrovirus*/*Epsilonretrovirus*-related (GE), and *Alpharetrovirus/Betaretrovirus*-related (AB). Placeholder groups (indicated by coloured squares) are reserved for ERVs that do not group within the diversity of established genera. Within these broad groups, additional subgroupings representing well-established monophyletic ERV lineages may be recognized. Here, some examples are indicated, shown emerging from each of their parent groups. Ultimately, some of these lineages might be attributed genus status, and would be moved to the appropriate level within this classification scheme

Since ERV sequences included in our classification scheme must by definition demonstrate phylogenetic links to exogenous retroviruses, it follows they can be integrated into a unified taxonomic scheme with a rational phylogenetic basis. This taxonomic scheme would provide the basis for assigning the ‘lineage’ component of the ID. Figure 4 illustrates a proposal for a unified scheme that integrates the classification of exogenous and endogenous retroviruses with minimal disruption to the existing schemas used for each. Within our proposed scheme, ERV loci should ideally be assigned IDs wherein the lineage component accurately reflects their position in such a unified schema. As discussed earlier, some ERVs exhibit phylogenetic and genomic characteristics that clearly identify them as endogenous representatives of contemporary virus groups (Table 1). However, the vast majority of ERVs fall outside the diversity defined by exogenous isolates. Thus, additional taxonomic groups would need to be created before the proposed nomenclature could be applied. These might be relatively broad to begin with—for example, the schema shown in Fig. 4 includes three ‘placeholder’ groups designed to act as temporary ‘bins’ for ERV loci that cannot be confidently placed within the existing taxonomic system approved by the ICTV. These groups correspond to three major divergences in orthoretroviral RT sequences [71], and are labelled as follows: *Spumavirus*-related (S), *Gammaretrovirus*/*Epsilonretrovirus*-related (GE), and *Alpharetrovirus/Betaretrovirus*-related (AB). Placeholder groups are reserved for ERVs that do not group within the diversity of established genera. Within these broad groups, additional subgroupings representing well-established ERV lineages can then be recognized. Wherever possible, ERVs should be assigned IDs that identify them at the level of individual lineages (i.e. monophyletic lineages of ERV sequences estimated to derive from a single germline colonisation event), or at the level of viral species for ERVs that show close relationships to exogenous viruses, such as some of those found in the mouse genome (see Table 2). Ultimately, some of the ERV lineages that lack exogenous counterparts might be recognised as fossil representatives of extinct lineages, and attributed genus status within the unified taxonomic scheme shown in Fig. 4.

With regard to the numeric ID component, each taxonomic level referenced by the nomenclature would require its own discrete numbering system, entirely independent of all other taxonomic levels, and within which numeric IDs are only assigned once. Inevitably, the taxonomic designations may be subject to a limited amount of change over time, since ERVs are often identified before their phylogenetic relationships are fully resolved. Similarly, the piecemeal task of identifying orthologs would be expected to cause ongoing adjustments to numeric IDs (e.g. as it becomes clear that an ERV in one species is orthologous to an ERV detected in another). Providing each adjustment generates a new key that is unique within the given taxonomic group, this can be accommodated.

Some ERV lineages have become known by particular names, and within these lineages, certain loci are also often known by particular numbers. We therefore propose that where ERV lineages or loci have established names or IDs that are well established and widely used, a mnemonic link to these should, where expedient, be retained. The examples shown in Table 2 illustrate how the proposed ID structure can support this.

The development of a consistent ERV nomenclature that uniquely identifies ERV loci would establish a basis for stably linking these loci to a wide range of relevant auxiliary information, such as cytogenetic location, or information about the genetic sub-structure of proviral insertions. This would compensate for the loss of such information from the ID itself, which would occur in some cases as a consequence of the standardization (see Table 2). Clearly, however, any auxiliary information attached to IDs would need to be collated and archived in a systematic way (i.e. using a database). Furthermore, ongoing maintenance of the nomenclature itself will be necessary, and a system of governance and oversight would need to be developed through which updates—e.g. addition, subtraction or merging of ERV loci, or reclassification of ERVs based on updated taxonomy—can be coordinated. An important aspect of nomenclature implementation will be the development of benchmarking procedures through which competing annotations can be assessed, as discussed more broadly for TEs in [72].

## Conclusions

In this review, we have provided an account of how ERV nomenclature has developed, identifying the idiosyncrasies that have been generated in current nomenclature systems as a consequence of their historical development. We propose a novel, rational approach to naming ERV loci that is designed to unambiguously identify individual ERV loci, while accommodating as far as possible the contingencies and idiosyncrasies of ERV annotation. In addition, the proposed system allows for seamless integration into existing schemes for classification of transposable elements and viruses [55, 63, 67, 69, 73].

## Abbreviations

ERV: endogenous retrovirus
LTR: long terminal repeat
NWM: New World monkey
OWM: Old World monkey
PBS: primer binding site
tRNA: transfer RNA
HERV: human endogenous retrovirus
MLV: murine leukemia virus
ICTV: International Committee for Virus Taxonomy
